# Imaging hot photocarrier transfer across a semiconductor heterojunction with ultrafast electron microscopy

**DOI:** 10.1073/pnas.2410428121

**Published:** 2024-09-26

**Authors:** Basamat S. Shaheen, Kenny Huynh, Yujie Quan, Usama Choudhry, Ryan Gnabasik, Zeyu Xiang, Mark Goorsky, Bolin Liao

**Affiliations:** ^a^Department of Mechanical Engineering, University of California, Santa Barbara, CA 93106; ^b^Department of Materials Science and Engineering, University of California, Los Angeles, CA 90095

**Keywords:** ultrafast electron microscopy, semiconductor heterojunction, interfacial transport, photocarrier dynamics

## Abstract

Semiconductor heterojunctions have gained significant attention for efficient optoelectronic devices owing to their unique interfaces and synergistic effects. Interaction between charge carriers with the heterojunction plays a crucial role in determining device performance, while its spatial-temporal mapping remains lacking. In this study, we employ scanning ultrafast electron microscopy (SUEM), an emerging technique that combines high spatial-temporal resolution and surface sensitivity, to investigate photocarrier dynamics across a Si/Ge heterojunction. Charge dynamics are selectively examined across the junction and compared to far bulk areas, through which the impact of the built-in potential, band offsets, and surface effects is directly visualized. In particular, we find that the heterojunction drastically modifies the hot photocarrier diffusivities in both Si and Ge regions due to charge trapping. These findings are further elucidated with insights from the band structure and surface potential measured by complementary techniques. This work demonstrates the tremendous effect of heterointerfaces on hot photocarrier dynamics and showcases the potential of SUEM in characterizing realistic optoelectronic devices.

## Significance

Semiconductor heterojunctions are crucial for optoelectronic devices. Despite the remarkable performance achieved, a complete understanding of the intricate interplay of the junction electrical potentials and charge transport phenomena across the heterojunction interface is missing. In particular, the “hot” photocarriers immediately after optical excitation play a crucial role in photovoltaic, photocatalytic, and photosensing devices, but their interaction with the heterojunction remains not understood. In this work, we apply scanning ultrafast electron microscopy to provide a holistic view of photoexcited charge dynamics in a Si/Ge heterojunction. We find that the built-in potential and the band offsets drastically modify the diffusion process of hot photocarriers across the heterojunction due to charge trapping, with significant implications for hot-carrier-based applications.

In many solid-state device applications, the creation of junctions within semiconductor materials provides a powerful approach to overcome the limitations of individual materials. For example, multijunction solar cells can harvest a wider spectrum of solar radiation by presenting multiple band gaps (1); electronic band alignments at semiconductor heterojunctions can be engineered to create quantum wells for tunable electronic and photonic applications (2); semiconductor heterojunctions show great promise in enhancing photocatalysis by balancing optical absorption and charge recombination properties of different materials (3); semiconductor heterojunctions also provide a platform for spintronics by joining magnetic and nonmagnetic semiconductors (4). Semiconductor junctions come in two types: homojunctions and heterojunctions. A homojunction occurs within a single semiconductor material with varying doping levels, whereas a heterojunction forms when two distinct semiconductor materials come into contact with each other (5). While p–n homojunctions are fundamental in semiconductor devices, semiconductor heterojunctions have attracted rapidly increasing research interests, where each side of the junction is made of a distinct semiconductor material with different compositions, bandgaps, crystal structures, or lattice constants (6–11). This diversity translates into a broader selection of materials to form heterostructures, unlocking complementary properties and synergistic effects to achieve superior device performance and opening up opportunities for innovative device concepts and applications across various fields.

The interface plays a crucial role in the functionality of a heterojunction device, far beyond serving as a divider between a homostructure device and a chemically dissimilar substrate (12). Often, the interface itself becomes the operational core of the device, shaping its characteristics and performance (13). Challenges in designing high-performance heterostructures, as outlined by Kroemer, revolve around understanding the energy band structure and elucidating charge transport phenomena across the interface (13). In particular, how energetic charge carriers injected either electrically or via photoexcitation interact with the heterojunction is the central factor that controls the device performance. Theoretical models can provide a starting point to estimate energy band offsets and built-in potentials for a heterojunction and predict charge transport behaviors accordingly (14–16). However, it has been a challenge to accurately simulate charge carrier dynamics in a mesoscopic structure, such as a heterojunction, where nonidealities associated with defects induced by processing limit the theoretical predictive power. Fundamentally, transport of highly nonequilibrium “hot” carriers across a heterojunction has not been fully modeled and understood (17). Experimentally, the influence of the heterojunction on charge transport is often inferred indirectly from electrical transport measurements (18), lacking the resolution to correlate local structures and potentials with charge behaviors. For these reasons, there exists a critical need for direct surface characterization techniques with sufficient space and time resolutions tailored to device-type structures, capable of yielding an accurate understanding of interface physics parameters and the complex charge dynamics near heterojunctions (19–22).

Time-resolved spectroscopy has been widely used to study charge transfer processes in heterojunctions occurring on an ultrafast time scale (23–27). Recently, transient microscopy techniques have been employed with high spatial-temporal resolution to observe charge dynamics localized across interfaces (28–31). While these methods provide valuable information, the large penetration depth of the optical probe obtains dynamical information mainly from the bulk (32). Surface-sensitive techniques such as X-ray photoelectron spectroscopy (XPS) and scanning Kelvin probe microscopy (SKPM) are capable of providing chemical information and high-resolution imaging of surface potential variations across interfaces, respectively (33–36). However, they usually lack the time resolution to directly map the charge dynamics on relevant time scales. Recently, time-resolved photoemission electron microscopy (TR-PEEM) has been used to probe charge transfer across a two-dimensional heterointerface with high temporal and spatial resolutions (37). Combining high spatial-temporal resolutions and high sensitivity to surface charge carrier dynamics, scanning ultrafast electron microscopy (SUEM) has emerged as a suitable tabletop method to visualize charge transfer across interfaces (38, 39). It utilizes a pulsed electron probe to scan the surface, eliciting secondary electrons (SEs) from the top few nanometers of materials (typically less than 10 nm). As a result, the contrast images obtained through SUEM, reflecting local changes in SE emission, capture photoinduced dynamic processes that influence the transport and emission of the SEs (40, 41). SUEM has been successfully applied to directly image photocarrier dynamics in a wide range of materials (42–48). Previously, SUEM was applied to study carrier dynamics in a silicon p–n homojunction (49) and a MoS_2_ homojunction (50), where the charge separation effect by the built-in potential at a homojunction was examined. Compared to a homojunction, heterojunctions can host more complex charge carrier dynamics due to the additional band offsets and different surface conditions on dissimilar materials forming the junction, whose impact on photocarrier transport remains less explored.

In this work, we apply SUEM to provide a holistic view of photoexcited charge dynamics in a Si/Ge heterojunction. The Si/Ge heterojunction samples fabricated through wafer bonding were chosen as our test model for this study due to the extensive research on both constituents and the wide range of applications for their heterojunctions (51, 52). By comparing SUEM contrast images taken at the heterojunction to those taken at bulk Si and Ge regions, we directly visualize the significant influence of the junction on photocarrier dynamics. In particular, we find that the built-in potential and the band offsets drastically change the hot photocarrier diffusivities on both Si and Ge side. Our results have significant implications on applications where efficient collection of hot photocarriers is desirable, such as hot-carrier-based photovoltaic (53) and photocatalytic (54) applications. Integrating our findings from SUEM with systematic XPS and SKPM characterizations allows us to draw a complete picture of photoexcited charge transport across the interface. Through this experimental surface characterization approach, we aim to achieve a better grasp and control of device interfaces, driving forward advances in semiconductor technology.

## 1. Results and Discussions

### 1.1. Quantifying the Heterojunction Parameters.

**Fig. 1.**
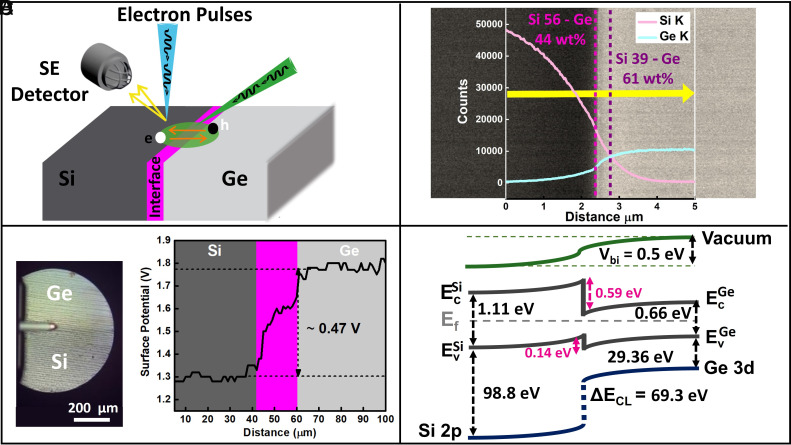
Schematic of the SUEM experiment and other characterizations of the sample. (*A*) Schematic illustration of the SUEM measurement of a Si/Ge heterojunction. SE: secondary electrons. (*B*) EDS mapping of the elemental composition across the junction interface. The X-ray counts corresponding to the Si K line and Ge K line are plotted against the position across the junction. The background is an SE contrast image taken in the same SEM prior to the EDS scan. Si region has a darker SE contrast compared to the Ge region. (*C*) Results of the scanning Kelvin probe microscopy across the Si/Ge heterojunction interface. The *Left* panel shows an optical image of the sample with the scanning probe. (*D*) Surface band diagram of the Si/Ge heterojunction determined from the XPS and SKPM measurements. $E_{C}$ and $E_{V}$ denote the conduction band edge and the valence band edge, respectively.

A schematic of our SUEM measurement is illustrated in Fig. 1*A*. More details are provided in *Materials and Methods*. Briefly, a cross-section of a Si/Ge heterojunction sample is examined using SUEM, which is an optical-pump-electron-probe technique. Optical excitations are generated in different regions of the heterojunction by an optical pump pulse (wavelength 515 nm, pulse duration 200 fs, beam diameter 30 μm, repetition rate 5 MHz, fluence 20 μJ/cm^2^). The optical response of the sample is monitored subsequently by a delayed electron pulse (30 keV kinetic energy, 30 to 40 electrons per pulse, pulse duration of a few picoseconds). The response is recorded as contrast images of SE emission from different locations of the sample surface.

Before the SUEM measurement, we conduct extensive characterizations of the Si/Ge heterojunction sample to gain necessary information to help interpret the SUEM results. More details are provided in Materials and Methods. A transmission electron microscope (TEM) image of the interface is shown in *SI Appendix*, Fig. S2, indicating an atomically smooth interface after bonding. Fig. 1*B* shows the elemental mapping based on energy dispersive spectroscopy (EDS) taken in a scanning electron microscope (SEM). X-ray counts corresponding to Si K line and Ge K line are measured across the junction. The elemental mapping suggests interdiffusion of Si and Ge atoms across the interface during the bonding process, making the Si/Ge interface a graded junction over a range of a few micrometers. Therefore, it is expected that the built-in potential ($V_{\mathrm{bi}}$) developed near the junction as a result of charge transfer will be spread across the lengthscale of a few micrometers. The background of Fig. 1*B* shows a representative SE contrast image of the Si/Ge heterojunction, where the Ge region exhibits a brighter contrast, signaling a higher SE yield. Given the primary beam energy at 30 keV, this difference in SE yield is likely due to the heavier atomic mass of Ge and the resulted larger elastic scattering cross-section (55).

To determine the crucial electrical properties of the heterojunction, including band offsets, built-in potential, and work functions, we further examined the sample using scanning Kelvin probe microscopy (SKPM) and X-ray photoemission spectroscopy (XPS). Both techniques, together with SUEM, are highly sensitive to the sample surface (top few nanometers) and, thus, reflect the junction properties as affected by the surface conditions, including surface band bending (56). SKPM measures the contact potential difference (CPD) between the sample surface and the scanning tip, which is determined by the difference in their work functions (57). Since the same scanning tip is used for measurement across the heterojunction, the corresponding CPD indicates the evolution of the work function across the junction. As shown in Fig. 1*C*, a higher CPD on the Ge side (∼0.5 eV higher than that on the Si side on average) is measured, suggesting a higher work function on the Ge side and a built-in potential across the junction $V_{\mathrm{bi}}\approx$ 0.5 eV. In addition, a smooth transition of the work function over a range of around 20 micrometers near the interface also suggests a graded junction, which is consistent with the EDS elemental mapping. XPS detects the binding energy of valence and core electrons as referenced from the Fermi level. XPS measurements of the relative positions between the valence band maximum (VBM) and specific core levels across a heterojunction can be used to estimate the band offsets at the junction (58, 59). Raw XPS spectra and the associated analysis are provided in *SI Appendix*, Fig. S1 and Note 1. From the analysis, the valence band offset between Si and Ge is determined to be 0.14 eV and the conduction band offset 0.59 eV. The complete surface band diagram of our Si/Ge heterojunction sample as determined by SKPM and XPS analyses is shown in Fig. 1*D*. Although Si/Ge heterostructure has been extensively studied, the majority of previous studies focused on Ge or Si_1−*x*_Ge_*x*_ thin films epitaxially grown on a Si or Ge substrate (51, 60). Different atomic structures and strain states resulting from different fabrication processes (e.g., epitaxial growth or wafer bonding) can make it difficult to directly compare the band alignments to the literature values. We also note here that the band diagram shown in Fig. 1*D* is based on surface-sensitive characterizations, where surface conditions can lead to electrical potential changes that can shift the band alignment. Surface effects can cause discrepancies with band diagrams obtained via bulk transport measurements (61). Since SUEM is also a surface-sensitive technique, the surface band diagram shown in Fig. 1*D* is more relevant for interpreting SUEM results.

### 1.2. Understanding SUEM Contrast in the Bulk Regions.

**Fig. 2.**
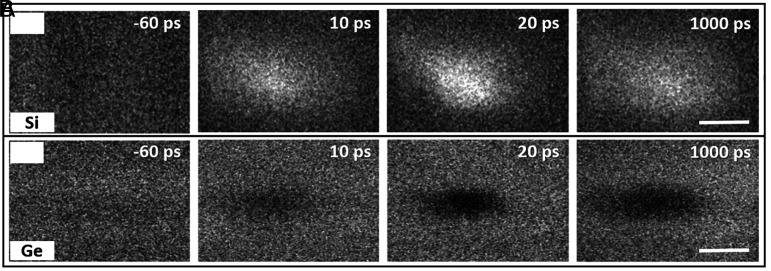
SUEM contrast images taken at bulk Si and Ge regions. (*A*) SUEM contrast images taken at bulk Si region far away from the junction. (*B*) SUEM contrast images taken at bulk Ge region far away from the junction. The time label indicates the delay time between the optical pump pulse and the electronic probe pulse. (Scale bar, 50 μm).

SUEM contrast images taken in Si and Ge bulk regions far away from the heterojunction are shown in Fig. 2. The time labels represent the delay time between the optical pump pulse and the electronic probe pulse. Negative times indicate the probe pulse arrives before the pump pulse. These contrast images are generated by subtracting a reference SUEM image taken at far negative times (−300 ps in our case) from SUEM images taken at later delay times. Therefore, the bright or dark contrasts seen in the SUEM images represent an increase or decrease in the SE yield as a result of the optical excitation. The lack of contrast at negative delay times in both Si and Ge bulk regions confirms that the sample has sufficient time to relax to its original state between two consecutive optical pump pulses (the pulses are separated by 200 ns). A recent SUEM study suggests that the SE collection field in vacuum between the sample surface and the SE detector can be modified by photocarrier excitation, which can lead to SUEM contrast at negative delay times (47). This effect is likely not significant in our study, due to the lack of contrast at negative times.

We observe bright SUEM contrast in Si bulk regions at positive delay times, suggesting an increased SE yield as a result of the optical excitation. The SUEM contrast mechanism in Si has been extensively investigated before (49, 56, 62, 63). In heavily doped Si, where surface band bending due to Fermi level pinning can significantly affect SE emission, photoinduced modification of the surface bands, the so-called “surface photovoltage” effect, can give rise to bright (dark) contrast in n-type (p-type) Si (56, 63). In undoped or lightly doped Si (as in this study), in contrast, optical excitation of photocarriers raises the average electron energy in the bulk, which leads to a higher SE yield and bright contrast (64). Therefore, the intensity of the bright contrast can be correlated to the local surface photocarrier concentration. Subsequent evolution of the bright contrast can be fitted to a Gaussian function, from which both the intensity and spatial extent of the photocarrier distribution can be extracted. The extracted intensity as a function of delay time in the Si bulk region is shown in *SI Appendix*, Fig. S3*A*. The initial rise of the intensity (∼7 ps) reflects the time resolution of the instrument while the later decay is due to the photocarrier recombination. In the bulk Si region, the fitted recombination time is around 2 ns, which is on the same order as the result of a previous transient reflectivity measurement on Si with a similar photocarrier concentration and surface condition (native-oxide-covered surface) (65). The evolution of the spatial distribution of the bright contrast represents the diffusion process of the photocarriers and will be analyzed later. Unlike Si, the Ge bulk region shows a dark contrast in the SUEM images, indicating a reduced SE yield as a result of photoexcitation. This behavior has previously been observed in GaAs (66), where the dark contrast was attributed to increased scattering of the SEs by the photoexcited carriers. Alternatively, the dark contrast could be caused by the surface band bending effect that can occur on Ge surfaces that are less sensitive to doping concentrations (67). In either case, the dark contrast can be correlated with the spatial distribution of photocarriers in Ge. The intensity of the Gaussian fit of the SUEM contrast is given in *SI Appendix*, Fig. S3*B*. In the bulk Ge region, the photocarrier recombination time is extracted to be roughly 6.3 ns.

### 1.3. Imaging Charge Transfer Across Si/Ge Heterojunction.

**Fig. 3.**
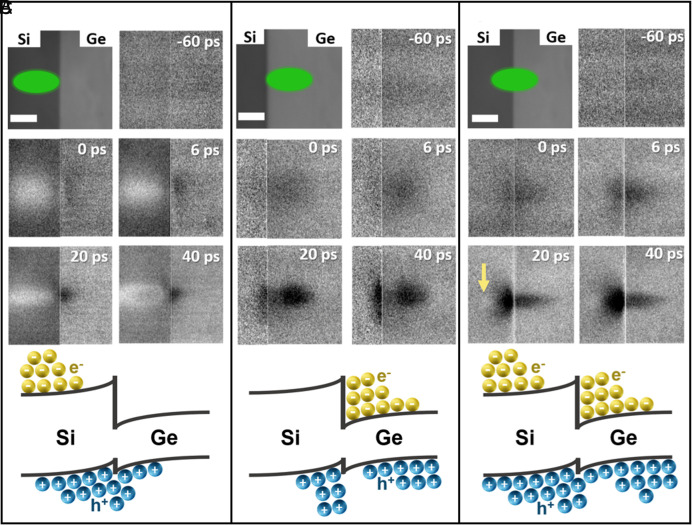
SUEM contrast images taken near the Si/Ge heterojunction. (*A*) SUEM contrast images showing photocarriers excited on the Si side diffusing into the Ge side. The *Top Left* panel is a static SEM image of the junction, where the green ellipse indicates the location of the optical pump beam. The *Bottom* panel shows a schematic of the photocarrier distribution as a result of the built-in potential and the band offsets of the heterojunction. (*B*) SUEM contrast images showing photocarriers excited on the Ge side diffusing into the Si side. (*C*) SUEM contrast images showing the dynamics of photocarriers excited right at the Si/Ge heterointerface. The yellow arrow highlights the emergence of bright contrast on the Si side away from the junction. (Scale bar, 50 μm).

Next, we use SUEM to directly visualize charge transfer across a Si/Ge heterojunction, as shown in Fig. 3. Complete datasets are provided as Movies S1–S3. Fig. 3*A* displays the scenario where the photocarriers are excited on the Si side (optical pump beam is indicated by the green ellipse in the static SEM image of the junction shown in the *Top Left* panel). As seen in the SUEM contrast images, initially after photoexcitation, bright contrast on the Si side is observed, which is consistent with the bulk Si result shown in Fig. 2. As the photoexcited carriers diffuse toward the junction, however, a contrast reversal occurs several micrometers away from the junction on the Si side. This effect is due to the built-in potential on the Si side (as shown in Fig. 1*D*) that attracts holes toward the junction while pushing the electrons away. The net effect is an accumulation of holes near the junction on the Si side, which leads to a reduced average energy of electrons and a lower SE yield (dark contrast). Some of the hot holes are able to migrate across the valence band offset into the Ge side, leading to dark contrast on the Ge side as well. Fig. 3*B* shows the opposite scenario, where the photocarriers are excited mostly on the Ge side. Due to the large conduction band offset (0.59 eV, as shown in Fig. 1*D*), most of the photoexcited electrons cannot migrate into the Si side. Although the built-in potential on the Ge side tends to push holes away from the junction, some of the holes photoexcited very close to the junction can transfer into the Si side and be trapped by the potential valley due to the valence band offset. This effect can be clearly seen in the SUEM images taken at 20 ps and 40 ps, where the holes on the Si side diffuse more quickly parallel to the junction, but much more slowly perpendicular to the junction.

Fig. 3*C* shows the case when the photocarriers are excited right at the junction. This case is analogous to the one studied by Najafi et al. in a Si p–n junction using SUEM (49), where the separation of photoexcited electrons and holes by the built-in potential was visualized. In our case, however, the presence of the heterojunction and the associated band offsets further complicates the photocarrier transport process. The built-in potential still tends to separate photoexcited electrons and holes on both sides of the junction, similar to that in a p–n homojunction. This is reflected in the emergence of a dark contrast on the Si side, where the built-in potential pushes electrons away from the junction while trapping the holes near the junction. At later delay times (20 to 40 ps), bright contrast emerges on the Si side tens of micrometers away from the junction (highlighted by a yellow arrow in Fig. 3*C*), indicating accumulation of electrons there as a result of the built-in potential separation effect. On the Ge side, this separation effect is not clearly seen because both electron and hole excitations lead to a dark contrast (Fig. 2*B*). However, a critical factor has so far been missing from the discussion. Given the high photon energy (2.4 eV) associated with the optical pump pulse, photoexcited electrons and holes are in a hot state immediately after excitation with very high electronic temperatures, which can lead to diffusivities much higher than the near-equilibrium values. This initial superdiffusion behavior of photocarriers has been observed in previous SUEM measurements of many semiconductors (42, 45, 46, 62). For example, in crystalline silicon, the initial diffusivity of photocarriers can be several orders of magnitude higher than the near-equilibrium values for up to 100 ps (62), while in cubic boron arsenide, the superdiffusion process persists for over 200 ps due to a hot phonon bottleneck effect (45). In all previous studies, superdiffusion of hot carriers was investigated in uniform semiconductors, whereas the interaction of hot photocarriers with structures such as heterojunctions remains less explored. Given the ubiquity of heterojunctions in optoelectronic applications, it is thus important to understand how hot photocarriers behave near heterojunctions.

### 1.4. Quantifying Hot Photocarrier Diffusion Near Si/Ge Heterojunction.

**Fig. 4.**
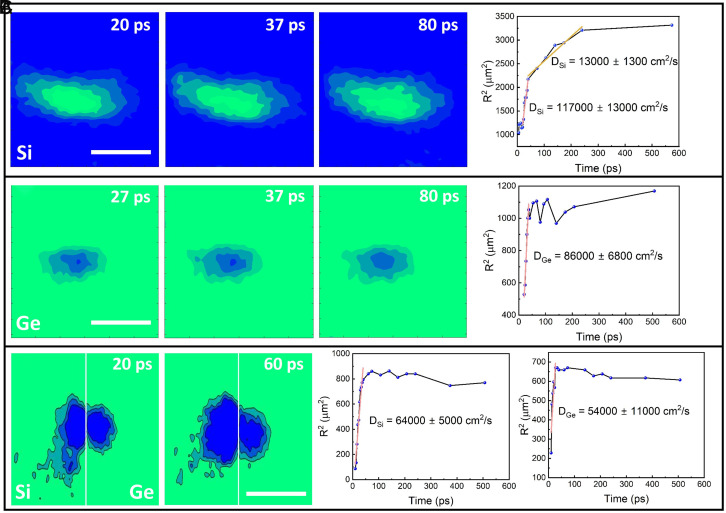
Diffusion of hot photocarriers excited near the Si/Ge heterojunction. (*A*) The *Left* three panels show contour plots of the SUEM contrast taken at bulk Si region far away from the junction as a function of delay time. The *Right* panel displays the squared radius of the photocarrier distribution as a function of delay time. (*B*) Similar plots as (*A*) for SUEM contrast images taken in bulk Ge region far away from the junction. (*C*) Similar plots as (*A*) for SUEM contrast images when photocarriers are excited at the heterojunction. The *Right* two plots show the squared radius of the photocarrier distribution as a function of delay time for the Si and Ge side of the junction, respectively. Orange solid lines show linear fits of the data to extract the hot photocarrier diffusivities. Uncertainty of the extracted hot photocarrier diffusivities is determined by 95% confidence level. (Scale bar, 50 μm).

In this section, we quantify the influence of the Si/Ge heterojunction on recombination and diffusion processes of hot photocarriers immediately after excitation from the SUEM contrast images. The fitted intensity of the photocarrier contrast near the Si/Ge heterojunction as a function of delay time is shown in *SI Appendix*, Fig. S3*C*, where the photocarrier recombination times in both Si and Ge regions are modified from those measured in bulk regions far away from the junction. This can be caused by a combination of the charge-separation effect of the junction built-in potential, which reduces the spatial overlap of electrons and holes and prolongs the recombination time, and the increased defect concentration due to mixing of Si and Ge near the junction (Fig. 1*B*), which can enhance defect-mediated recombination. Stages of photocarrier diffusion in uniform bulk Si have been visualized and analyzed by SUEM in a previous work (62). Here, our observation in the bulk Si region (as shown in Fig. 4*A*) is similar to the previous report. The first three panels in Fig. 4*A* show contour plots with constant SUEM contrast intensity as a function of delay time in the bulk Si region. Tracking the spatial-temporal evolution of the contours can provide quantitative information about the photocarrier diffusion process. Alternatively, the SUEM contrast images can be fitted to 2D Gaussian functions with a time-dependent radius, which is shown in the fourth panel of Fig. 4*A*. The slope of the squared radius of the Gaussian distribution as a function of delay time represents the diffusivity at a given moment. It can be clearly seen that photoexcited carriers diffuse out rapidly right after photoexcitation for a time period of about 100 ps, before slowing down and eventually approaching the near-equilibrium ambipolar diffusivity value of about 20 cm^2^/s. Our result in the bulk Si region agrees qualitatively with the previous SUEM experiment on heavily doped Si (62). In the bulk Ge region (Fig. 4*B*), we observed a similar hot photocarrier diffusion regime with a slightly lower hot photocarrier diffusivity in the first 100 ps compared to that in Si. Although both electrons and holes in Ge have higher near-equilibrium mobilities (and thus diffusivities) than those in Si, a few factors can possibly lead to slower hot carrier diffusion. First, Ge has a smaller band gap (0.66 eV) compared to Si (1.11 eV), suggesting electrons and holes can be excited by the optical pump (2.4 eV) to higher-energy bands with heavier mass and lower mobility. Second, surface bands are observed to play a significant role in pinning the Fermi level on the Ge surface regardless of doping concentration (67), suggesting a higher surface defect concentration that can scatter the photocarriers and slow down their diffusion.

Fig. 4*C* shows the photocarrier diffusion process near the Si/Ge heterojunction when the photocarriers are excited right at the interface. Influenced by the built-in potential near the junction, photocarrier diffusion on both sides becomes more anisotropic than the bulk regions. Moreover, the hot carrier diffusivities in both Si and Ge regions near the heterojunction are significantly suppressed. On the Si side, mainly the diffusion of the photoexcited holes is imaged due to the charge separation effect of the built-in potential as discussed in the previous section. The hot carrier diffusion on the Si side is slowed down, likely due to the built-in potential that tends to trap holes near the junction. Similarly, on the Ge side, mainly the hot photocarrier diffusivity of electrons is imaged due to charge separation, which is suppressed due to the charge trapping effect of the heterojunction (Fig. 3*C*). The observed suppression of hot photocarrier diffusion due to charge trapping by the heterojunction suggests that, in applications where collection of hot photocarriers is essential, such as hot-carrier-based photovoltaics (53), photodetection (68), and photocatalysis (54), heterojunctions need to be carefully designed to avoid the charge trapping effect that might negatively impact the hot-carrier collection efficiency. Furthermore, this direct visualization of hot photocarrier dynamics near a heterojunction enabled by the development of SUEM can provide an experimental basis to benchmark photocarrier transport theories and simulations, which can lead to better understanding and design of optoelectronic devices based on semiconductor heterojunctions.

### 1.5. Conclusion.

In summary, we use SUEM to directly visualize photocarrier dynamics near a semiconductor heterojunction in space and time. The unique combination of space and time resolutions and surface sensitivity of SUEM allows us to explore the interaction between photoexcited charge carriers and the junction built-in potential, band offsets, and the surface effects, assisted by other characterization techniques that provide comprehensive parameters of the Si/Ge heterojunction under study. In particular, we observe the impact of the heterojunction on the initial diffusion of hot photocarriers excited right at the interface, which can have significant implications for hot-carrier-based photovoltaic, photocatalysis, and photodetection devices. Our study also showcases SUEM as an emerging technique with the potential to image charge-transport processes in realistic devices.

## 2. Methods

### 2.1. Sample Preparation.

(001) Germanium was bonded to (001) silicon using EVG^®^ ComBond^®^ equipment under high vacuum (10^−8^mtorr) and room temperature. This process is similar to other reported surface-activated bonding (SAB) or in situ sputtering techniques, in which Ge and Si surfaces are sputtered with an Ar ion beam (300 eV) at a shallow angle of 45 degrees to remove unwanted native surface oxides prior to bonding (69, 70). The post Ar-beam-treated samples are placed face-to-face and pressure is applied to initiate the bond. The Si crystal (thickness of 700 μm, 1 × 1.5 cm) is lightly doped by boron with an estimated p-type concentration below 10^16^ cm^−3^. The Ge crystal (thickness of 700 μm, 1 × 1.5 cm) is lightly doped by antimony with an estimated n-type concentration below 10^16^ cm^−3^.

### 2.2. Sample Characterization.

Structural characterization for Si/Ge heterojunction was conducted using electron microscopy techniques including high-resolution scanning transmission electron microscopy (STEM) and energy dispersive X-ray spectroscopy (EDX). An FEI Nova 600 Nanolab Dual Beam SEM/FIB was used to prepare cross-section TEM samples roughly 100 nm thick using a Ga source and then transferred to a TEM grid using a standard lift-out procedure. High-resolution transmission electron microscopy (HRSTEM) of the interface was taken with a Cs-corrected JEOL GrandARM at 300 kV. Asylum Research Jupiter Atomic Force Microscopy (AFM) was used in scanning Kelvin probe mode (SKPM) to measure the surface potential on the Si–Ge cross-section sample with a scan angle of 90 degrees using a silicon tip with Ti/Pt coating.

In order to determine the energy band offsets at Si–Ge interface, Kraut’s method was followed in XPS analysis (58, 59). Thermo Fisher Escalab Xi+ XPS Microprobe with a monochromated Al Kα X-ray source (beam energy of 1486.7 eV) was used to measure the binding energy associated with core levels (CLs) and valence band maxima (VBM). The XPS spectra were collected from three locations on the Si/Ge cross-section: 1) the bulk Si region to measure the CL binding energy of Si 2p and the VBM; 2) the bulk Ge region to measure the CL binding energy of Ge 3d and the VBM; 3) the Si–Ge heterojunction region to measure the CL binding energy of Si 2p and Ge 3d at the interface. In order to neutralize positive charge accumulation on the surface and overcome the shift in binding energy, charge compensation by an electron flood source was used. Besides, all the binding energy values were corrected by shifting the carbon 1s CL peak to 285 eV.

### 2.3. SUEM.

The detailed description of the SUEM setup was provided in our previous publications (44, 45). Briefly, a fundamental infrared (IR) laser (Clark MXR IMPULSE) operating at a central wavelength of 1,030 nm with a pulse duration of 150 fs is directed to frequency-doubling crystals to create the visible pump beam (wavelength 515 nm) and the ultraviolet (UV) photoelectron excitation beam (wavelength 257 nm). The UV excitation beam is directed through the column of an SEM (Thermo Fisher Quanta 650 FEG) and onto the apex of a cooled Schottky field emission gun (ZrO_2_-coated tungsten) to generate electron pulses with picosecond durations via the photoelectric effect. The photogenerated electron pulses are accelerated inside the SEM column to 30 keV kinetic energy. The visible pump beam is directed inside the microscope to initiate the excitation of the sample. The diameter of the optical pump beam used in this study is around 30 μm. The distance traveled by the visible pump beam is adjusted by a computer-controlled mechanical delay stage (Newport DL600, delay time range −0.7 to 3.3 ns). Time zero in our SUEM experiments was determined by fitting the rising dynamics (the intensity of the increasing contrast) near time zero to an error function, $1+\mathrm{erf}[\left(t-t_{0}\right)/\tau ]$, where τ is the rise time and $t_{0}$ is the fitted time zero. The fitted $t_{0}$ values across different datasets are within 2 ps. All experiments reported here were conducted at a laser repetition rate of 5 MHz, an electron probe beam current of 30 to 40 pA, and a visible pump beam fluence of 20 μJ/cm^2^. This particular optical fluence was chosen to achieve the highest signal-to-noise ratio while avoiding permanent optical damage of the sample at higher fluences. Considering the near 45^°^ incident angle of the optical pump beam, the excited photocarrier concentration is estimated to be around $5\times 10^{17}$ cm^−3^ in Si and $1\times 10^{19}$ cm^−3^ in Ge based on their documented optical properties. The probe beam current corresponds to 30 to 40 electrons per pulse and a pulse width of a few ps (45). The images were acquired using a dwell time of 300 ns per pixel and an integration of 256 frames. The samples were mounted on an SEM cross-section stub to measure the interface as received after bonding without polishing or other surface treatment.

## Supplementary Material

Appendix 01 (PDF)

Movie S1. SUEM contrast image series when the photoexcitation is on the Si side of the junction.

Movie S2. SUEM contrast image series when the photoexcitation is on the Ge side of the junction.

Movie S3. SUEM contrast image series when the photoexcitation is at the Si/Ge hetero-interface.

## Data Availability

Raw SUEM images are provided in the Movies S1–S3. All other data are included in the manuscript and/or supporting information.
