# Supplementary material for: Imaging hot photocarrier transfer across a semiconductor heterojunction with ultrafast electron microscopy
Source: Proc Natl Acad Sci U S A. 2024 Sep 26;121(40):e2410428121. doi: 10.1073/pnas.2410428121 (PMC11468150; doi:10.1073/pnas.2410428121)
Supplement: Supplementary file 1 — Appendix 01 (PDF) [file pnas.2410428121.sapp.pdf]

Supplementary Materials: Imaging Hot Photocarrier Transfer across a Semiconductor Heterojunction with Ultrafast Electron Microscopy

Basamat Shaheen,[1] Kenny Huynh,[2] Yujie Quan,[1] Usama Choudhry,[1]
Ryan Gnabasik,[1] Zeyu Xiang,[1] Mark Goorsky,[2,*] and Bolin Liao[1,†]

[1]*Department of Mechanical Engineering,*
University of California, Santa Barbara, CA 93106, USA
[2]*Department of Materials Science and Engineering,*
University of California, Los Angeles, CA 90095, USA

(Dated: August 9, 2024)

* goorsky@seas.ucla.edu
† bliao@ucsb.edu

SUPPLEMENTARY TEXT:

I. ANALYSIS OF THE HETEROJUNCTION POTENTIALS BASED ON XPS

X-ray photoemission spectroscopy (XPS) is a widely used surface-sensitive technique to determine band offsets and built-in potentials across heterojunctions. XPS directly measures the binding energy, which is the energy difference between the Fermi level and a selected core level. Valence band offsets at a heterojunction can be determined by Kraut's method [1, 2], where the valence band offset can be determined by conducting XPS measurements in bulk regions on both sides and at the interface. Raw XPS data measured from our Si/Ge heterojunction sample is given in Fig. S1. The valence band offset is given by [1, 3]:

$$\Delta E_V = (E_{CL1} - E_{V1})_{\text{bulk1}} - (E_{CL2} - E_{V2})_{\text{bulk2}} - (E_{CL1} - E_{CL2})_{\text{interface}}, \tag{1}$$

where ΔE_V is the valance band offset, $(E_{CL1}-E_{V1})_{\text{bulk1}}$ and $(E_{CL2}-E_{V2})_{\text{bulk2}}$ are binding energy differences between a chosen core level and the valence band maximum (VBM) measured in bulk regions on both sides of the heterojunction, respectively, and $(E_{CL1} - E_{CL2})_{\text{interface}}$ is the binding energy difference of the two core levels measured at the heterojunction interface. In our case, Si $2p$ and Ge $3d$ are chosen as the core levels. The corresponding binding energy differences are labeled in Fig. S1. Based on the XPS data, the valence band offset is calculated to be 0.14 eV. Then, the conduction band offset ΔE_C can be determined by [3]:

$$\Delta E_C = \Delta E_V + (E_{g1} - E_{g2}), \tag{2}$$

where E_{g1} and E_{g2} are the bandgaps of the two materials forming the junction. Given the Si bandgap of 1.11 eV and the Ge bandgap of 0.66 eV, the conduction band offset ΔE_C is calculated to be 0.59 eV. Additionally, given the VBM binding energy difference measured in the bulk Si and Ge regions (∼0.36 eV), the total built-in potential across the heterojunction can be estimated to be 0.5 eV. The band diagram based on this analysis is given in Fig. 1d in the main text.

II. DATA FITTING

A. Contrast Radius Fitting

The SUEM contrast images in bulk Si and Ge are fitted to Gaussian functions with a time-dependent radius and intensity. The horizontal and vertical radii are determined by fitting the data averaged within the 30-pixel thick ribbons cut along the y-axis and x-axis, respectively. Fig. S4(a) shows the example of the area selected for the Gaussian fitting, and Fig. S4(b) and (c) show the corresponding fitting results. The horizontal radius at the heterojunction is extracted from the contour plot, where the distance is measured between the center and the edge of the outer region with a certain threshold.

B. Diffusivity Fitting

The diffusivity D is fitted using the equation [4]:

$$R^2 = 4Dt, \tag{3}$$

where R is the radius of the photocarrier distribution, and t is the time delay. The linear fitting of the squared radius and the fitted diffusivity are shown in Fig. 4 in the main text.

SUPPLEMENTARY FIGURES

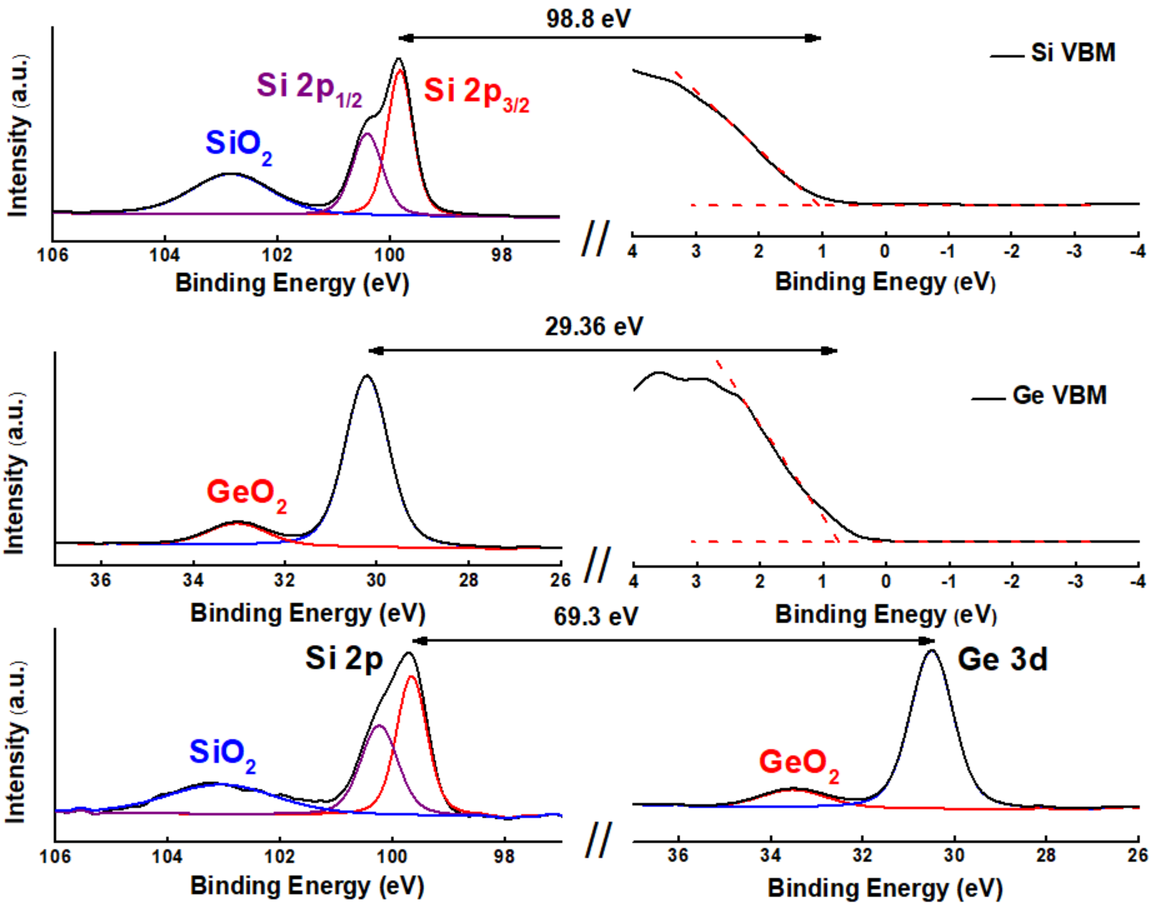

FIG. S1. **XPS spectra measured across the Si/Ge heterojunction.** The top panel shows the XPS spectrum measured in the Si bulk region, where the peak corresponding to the Si $2p$ level and the continuum corresponding to the valence band maximum (VBM) are labeled. The middle panel shows the XPS spectrum measured in the Ge bulk region, where the peak corresponding to the Ge $3d$ level and the continuum corresponding to the VBM are labeled. The lower panel shows the XPS spectrum measured at the heterojunction interface, where the peaks corresponding to the Si $2p$ level and the Ge $3d$ level are labeled.

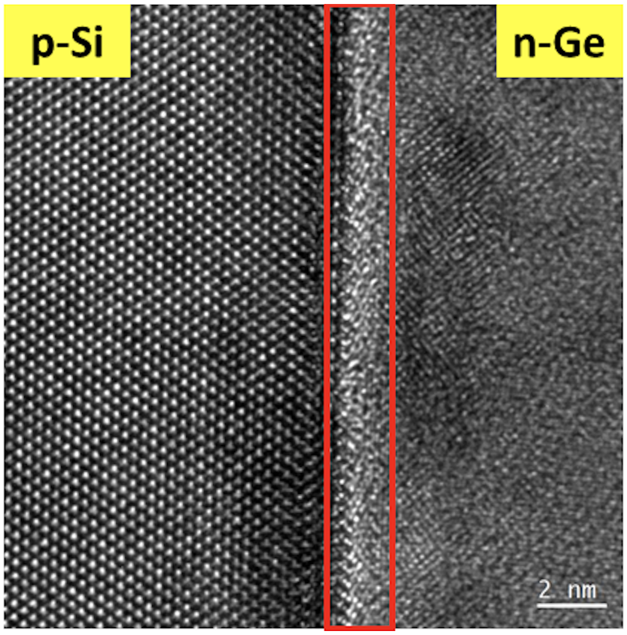

FIG. S2. TEM image of the Si/Ge heterojunction after wafer bonding.

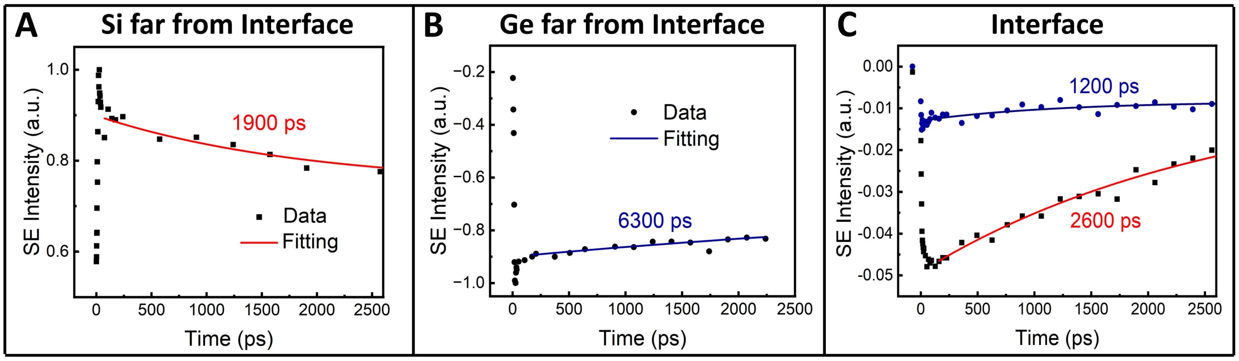

FIG. S3. The intensity of the SUEM contrasts as a function of delay time taken in (a) the bulk Si region far from the junction, (b) the bulk Ge region far from the junction, and (c) the Si side (red line) and Ge side (black line) of the heterojunction. The intensities are extracted by fitting the raw SUEM contrasts to 2D Gaussian functions with time-dependent intensity and radius.

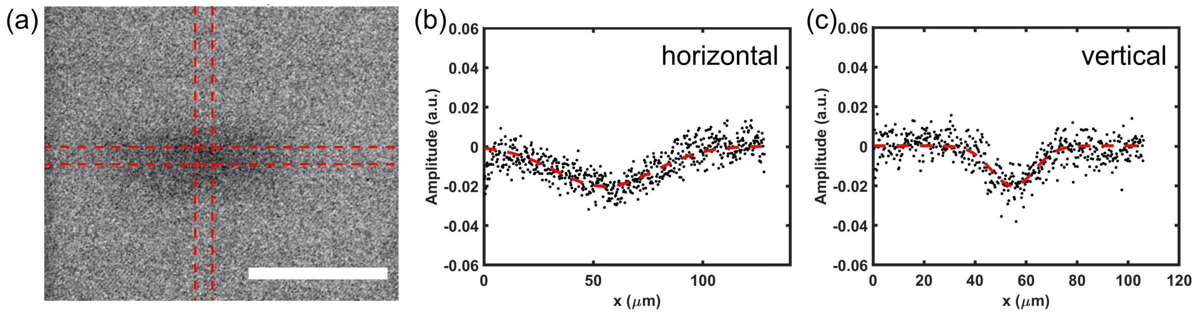

FIG. S4. The SUEM contrast image of Ge at $t = 30\,\mathrm{ps}$ and the corresponding fittings. (a) The dashed red lines show the selected horizontal and vertical radii fitting areas. (b) Gaussian fitting of the horizontal radius. (c) Gaussian fitting of the vertical radius.

CAPTIONS FOR SUPPLEMENTARY MOVIES

Supplementary Movie S1: SUEM contrast image series when the photoexcitation is on the Si side of the junction.

Supplementary Movie S2: SUEM contrast image series when the photoexcitation is on the Ge side of the junction.

Supplementary Movie S3: SUEM contrast image series when the photoexcitation is at the Si/Ge hetero-interface.

Supplementary References

---

[1] E. Kraut, R. Grant, J. Waldrop, and S. Kowalczyk, Precise determination of the valence-band edge in x-ray photoemission spectra: application to measurement of semiconductor interface potentials, Physical Review Letters **44**, 1620 (1980).

[2] E. Kraut, R. Grant, J. Waldrop, and S. Kowalczyk, Semiconductor core-level to valence-band maximum binding-energy differences: Precise determination by x-ray photoelectron spectroscopy, Physical Review B **28**, 1965 (1983).

[3] S. A. Chambers, L. Wang, and D. R. Baer, Introductory guide to the application of XPS to epitaxial films and heterostructures, Journal of Vacuum Science & Technology A **38** (2020).

[4] E. Najafi, V. Ivanov, A. Zewail, and M. Bernardi, Super-diffusion of excited carriers in semiconductors, Nature Communications **8**, 15177 (2017).
